# A Complete Record from Colonization to Extinction Reveals Density Dependence and the Importance of Winter Conditions for a Population of the Silvery Blue, *Glaucopsyche lygdamus*

**DOI:** 10.1673/031.011.13001

**Published:** 2011-10-03

**Authors:** Matthew L. Forister, James A. Fordyce, Andrew C. McCall, Arthur M. Shapiro

**Affiliations:** ^1^ Program in Ecology, Evolution and Conservation Biology, Department of Biology, University of Nevada, Reno; ^2^ Department of Ecology and Evolutionary Biology, University of Tennessee, Knoxville; ^3^ Department of Biology, Denison University, Granville, Ohio; ^4^ Center for Population Biology, University of California, Davis

**Keywords:** exotic host, Lycaenidae, population dynamics

## Abstract

Butterflies in the family Lycaenidac are often the focus of conservation efforts. However, our understanding of lycaenid population dynamics has been limited to relatively few examples of long-term monitoring data that have been reported. Here, factors associated with population regulation are investigated using a complete record of a single population of the silvery blue, *Glaucopsyche lygdamus* Doubleday (Lepidoptera: Lycaenidae). Adults of *G. lygdamus* were first observed in an annual grassland near Davis, California, in 1982 and were last seen in 2003. Relationships between inter-annual variation in abundance and climatic variables were examined, accounting for density dependent effects. Significant effects of both negative density dependence and climatic variation were detected, particularly precipitation and temperature during winter months. Variation in precipitation, the strongest predictor of abundance, was associated directly and positively with butterfly abundance in the same year. Winter temperatures had a negative effect in the same year, but had a lagged, positive effect on abundance in the subsequent year. Mechanistic hypotheses are posed that include climatic effects mediated through both larval and adult plant resources.

## Introduction

There is a long tradition in ecology of debating the importance of intrinsic factors (e.g., negative density dependence) versus extrinsic factors (e.g., mortality associated with weather) in determining variation in the abundance of organisms (Andrewartha and Birch 1973; Benton et al. 2006). Many researchers now favor a perspective that asks under what conditions one or the other type of process might be more prevalent, and when they might interact (e.g. Ziebarth et al. 2010). Hundreds of relevant datasets exist, though relatively few of these involve butterflies, and even fewer involve butterflies in the family Lycaenidae (Ehrlich et al. 1972; Hochberg et al. 1992; Guiney et al. 2010). Given their specialized life histories (e.g., mutualistic interactions with ants), short life spans, and patchy population structure, lycaenid population dynamics might differ in important ways from other butterflies. Since lycaenids comprise a large fraction of Lepidoptera that are legally protected (New 1993), there is an inherent interest, from a conservation perspective, in developing a better understanding of the population dynamics of lycaenids.

Here we investigate the history of a single population of the silvery blue, *Glaucopsyche lygdamus* Doubleday (Lepidoptera: Lycaenidae). *G. lygdamus* is obligately univoltine, flying in early spring at low elevation in Northern California (Shapiro and Manolis 2007). Larvae complete feeding and pupate by late spring. Pupae remain in diapause overwinter, with adults eclosing typically in March. This species colonized an area near Davis, California in 1981 (adults first observed in the spring of 1982) and was last seen in 2003. Prior to 1981, the species was known briefly from an area north of Davis in the early 1970s, but otherwise unknown from the immediate region until it colonized the focal location of this study. Other populations are known within approximately 50 km of this site. The dataset examined here is considered complete, in that it encompasses the colonization and extinction of a population. Thus the data is useful for addressing the relative importance of climatic factors and density dependence in determining population fluctuation, persistence, and extinction.

## Materials and Methods

The focal location for this study was the intersection of 1–80 and Old Davis Road (38.53 N° 121.76° W), south of Davis, California; an annual grassland that includes an exotic larval host of *G. lygdamus*, hairy vetch, *Vicia villosa* Roth (Fabales: Fabaceae) (Graves and Shapiro 2003). The use of exotic hosts by *G. lygdamus* has increased dramatically during the past 60 years, as native hosts from the genera *Lathyrus* and *Lupinus* have become rare throughout much of the Central Valley (Shapiro and Manolis 2007).

One of the authors, AM Shapiro, had been monitoring the focal site phenologically since 1974. After *G. lygdamus* adults were observed in 1982, the site was monitored approximately every 3–4 days during the flight season for an average of 8.5 visits per year; monitoring has continued following extinction in 2003. At each visit, a fixed transect was walked and the number of adult individuals seen was recorded. For our purposes here, “abundance” is the total number of individuals observed in one year, which corresponds to one flight season. One possible bias in such a census technique could result from adult butterflies occurring in different parts of the landscape in different years, perhaps spatially tracking nectar resources. However, the fixed transect was designed to be extensive relative to the distribution of potential resources that could attract the butterflies.

Climatic data was collected from a Davis weather station: #2294 in the National Weather Service Cooperative Observer Program. Weather data for maximum and minimum daily temperatures as well as for precipitation totals was summarized on both an annual (fall-summer) and a seasonal basis (December-February, March-May, JuneAugust, September-November).

Analyses were conducted in two phases to investigate associations between climatic variables and abundance while accounting for density dependence. First, all seasonal weather variables (average daily maximum temperature, average daily minimum temperature, precipitation) from the current and previous year as well as abundance in the previous year were included in multiple regression models, with change in abundance relative to the previous year as the dependent variable. “Year” does not refer to a calendar year, but to a biological year from SeptemberAugust. More specifically, these models investigated the following predictor variables: average daily minimum and maximum temperatures and precipitation from fall, winter, spring, and summer of year t-1; average daily minimum and maximum temperatures and precipitation from fall, winter, and spring of year t (summer of the current year was not included as adults are not observed past spring); and abundance in year t-1. The inclusion of weather variables from the previous biological year in analyses is justified by the biology of *G. lygdamus* as described above. Davis has a Mediterranean climate in which precipitation typically falls only from October to April. Rainfall in one rainy season thus might affect host plant growth in the subsequent spring, which might then affect the abundance of adults whose larvae fed on those plants the spring after that. One-year time lags could easily occur, e.g., with the demographic impact of abiotic conditions skipping a year. The dependent variable for these models was not raw abundance. Rather, ΔN was examined, which is the change in abundance from the previous year, t-1, to the current year, t. Thus, the first year for analyses was 1983, the year after adults were first observed; the last year for analyses was the year after the last adult had been recorded. Using this metric of change in abundance (ΔN = N_t_ - N_t-1_) is one of many approaches that have been employed in studies of density dependence, which should be apparent as a negative association between N_t-1_ and ΔN_t_ (Ziebarth et al. 2010). In addition to the inclusion of ΔN in multiple regression models, we have also employed the randomization test of Pollard et al. (1987) for detecting density dependence (Turchin 1995).

All possible models involving the three weather variables from seven seasons—all four seasons in the previous year and three in the current—as well as abundance in the previous year were fit to the data. Up to eight independent variables were allowed in individual models. Models were subsequently ranked based on Akaike information criterion scores corrected for small sample sizes (AICc) (Sugiura 1978). Assumptions of multiple regression were checked, including normality of residual error and collinearity of predictors, which was evaluated using variance inflations factors. These were not checked for all possible models, but were investigated for the subset of best-fitting models as judged by Akaike information criterion scores.

Following multiple regression analyses, specific weather variables were identified as having the strongest associations with abundance, and used in subsequent path analyses, or structural equation models. Structural equation modeling is useful in comparing models with different combinations of connections among variables, and differs from multiple regression in accounting for relationships among predictor variables and including multiple dependent variables (Shipley 2000; Grace 2006). These models included the subset of weather variables identified from multiple regression models, as well as abundance in the previous year (N_t-1_) and the change in abundance from one year to the next (ΔN). By exploring connections among these variables with structural equation modeling, we were able to verify and visualize complex relationships between variables across years. Structural equations were modeled using AMOS version 5 (IBM, http://www-01.ibm.com/software/analytics/spss/products/statistics/amos/), and multiple regression analyses were performed in JMP version 8 (JMP, www.jmp.com).

**Figure 1.**
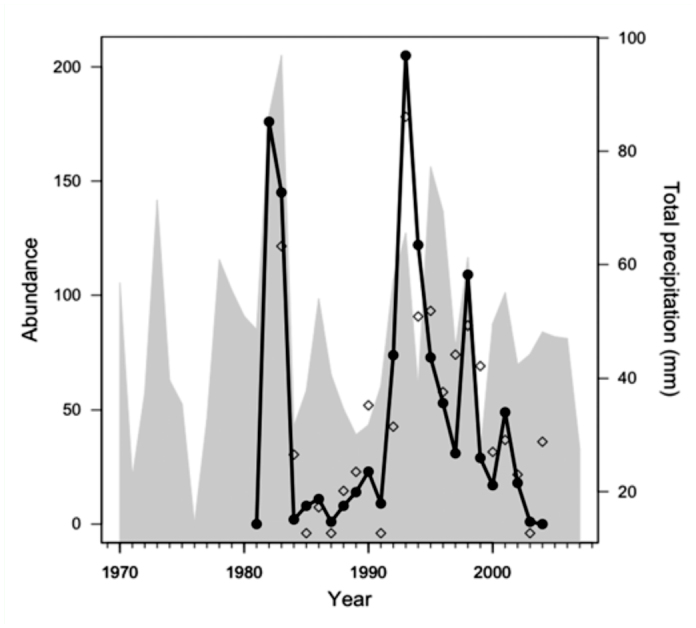
Annual variation in abundance (solid line) of adult silvery blue (*Glaucopsyche lygdamus*) butterflies at Old Davis Road and annual total rainfall (gray shading). Open diamonds are predicted points from the best-fitting multiple regression model (see Table 1); at points where the model predicts extinction, the points are shown offset below zero (i.e., 1991). High quality figures are available online.

## Results

The best-fitting multiple regression models examining weather and abundance are shown in Table 1. Judging by the standardized beta coefficients associated with each variable, as well as the consistency of variable inclusion in the best models, the most powerful predictors were abundance in the previous year (N_t-1_), average daily maximum temperature for the previous winter, precipitation in the current winter, and average daily minimum temperature in the current winter. These same weather variables retain their importance in lower-ranked models, which are not shown. Among those predictors, annual variation in winter precipitation tended to have the strongest association with abundance; variation in precipitation is illustrated in Figure 1 along with variation in adult abundance and fitted values from the bestfitting multiple regression model. The importance of density dependence was supported by results from a randomization test: the observed correlation between N_t_-1 and ΔN was -0.56 (*p* < 0.01; 10,000 permutations) (Pollard 1987).

**Figure 2.**
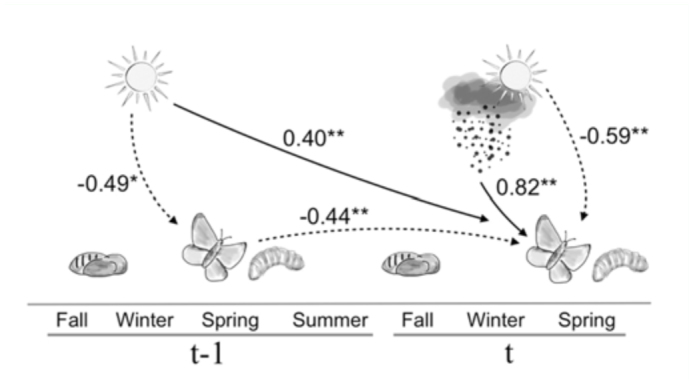
(a) Illustration of *Glaucopsyche lygdamus* life cycle (relative timing of adult, larval, and pupal stages) and path model relating change in adult abundance at time t (relative to the previous year) to the following weather variables: winter average daily maximum temperature in year t-1; winter precipitation as well as winter average daily minimum temperature in year t. Adult abundance at t-1 is the count of individuals in each spring, while variation in abundance in year t is expressed as ΔN, or the change in abundance from t-1 to t (see text for more details). Path coefficients are shown with dashed lines for the negative coefficients (*p < 0.05; **p < 0.01). R^2^ for N_t-1_ and ΔN in this model are 0.25 and 0.81, respectively. Also, see Table 1 for results from a multiple regression model including the same variables. High quality figures are available online.

**Table 1.**
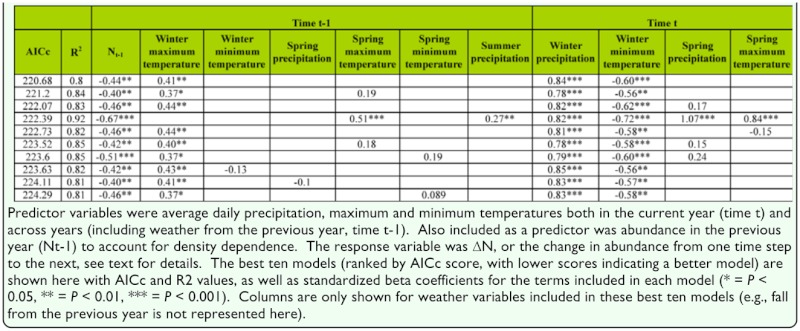
Results from multiple regression models investigating the association between variation in abundance and weather variables.

**Table 2.**
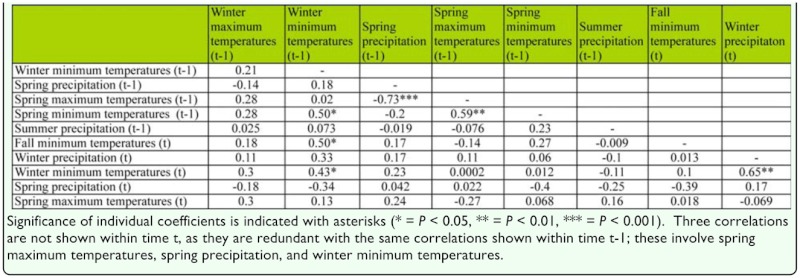
Pearson product-moment correlation coefficients between all weather variables shown in Table 1.

Multiple regression models are, of course, sensitive to highly correlated predictor variables. However, variance inflation factors were low—rarely greater than five— suggesting that these results are not biased by multicollinearity. Furthermore, Table 2 presents simple correlations among the subset of weather variables from the best models; correlations among predictors are generally but not always low.

Following the results from initial analyses, subsequent analyses using structural equation modeling focused on abundance in the previous year, change in abundance in the current year relative to the previous year (ΔN), and the three weather variables mentioned above as most predictive in multiple regression models (the abundance variables are endogenous, the weather variables are exogenous). Figure 2 is an illustration of one possible path model involving these variables. Although containing all of the same variables as the best multiple regression model (Table 1), the model shown in Figure 2 allows us to additionally consider both the direct and indirect connections between temperatures in the previous winter and change in abundance in the current year. On the one hand, there is a negative association between previous winter temperature and abundance in that year (path coefficient -0.49, Figure 2). However, combined with negative density dependence (-0.44), this results in an indirect positive effect of average daily maximum temperatures from the previous year. After accounting for that indirect effect, the model suggests an additional, direct positive effect of previous temperatures (path coefficient 0.40).

The model shown in Figure 2 was compared to two other models. The first had all of the same paths as in Figure 2 minus the connection between previous winter temperature and abundance in the previous year. The second model had the paths shown in Figure 2 minus the direct connection between previous winter temperature and ΔN. Judging by Akaike information criterion scores, the model shown in Figure 2 had the best fit to the data: 27.03 for the model in Figure 2, 30.69 for the model lacking the direct connection between previous temperature and ΔN, and 34.42 for the model without the path between previous winter temperature and abundance in the previous year. Although not illustrated in Figure 2, all models included correlations between weather variables.

## Discussion

The relationships that are reported here between abundance and weather variables involve potentially complex direct and indirect effects, likely mediated through different life history stages (Kingsolver 1989). One of the more pronounced positive associations is between winter precipitation and a change in abundance (ΔN) in the current year relative to the previous year (Table 1, Figure 2). *G. lygdamus* caterpillars have completed development by late spring, and are pupae throughout the winter (see life cycle illustration in Figure 2). Thus the positive effect of precipitation is not an effect of enhanced growth of larval hosts. Instead, the positive relationship between precipitation and the numbers of butterflies observed in the spring could be driven by a number of factors, including densities of natural enemies such as parasitoids, which might be depressed by winter rains resulting in higher larval and pupal survival. Another possibility is that winter precipitation has a positive effect on the availability of nectar resources in the spring, which could be associated with longer adult life spans and reduced emigration from the site. Both increased life span and reduced emigration could result in greater numbers of butterflies observed. The availability of nectar could also influence population dynamics more generally by enhancing the fecundity of females (Murphy et al. 1983; O'Brien et al. 2004), leading to a density dependent effect on the following year.

The effects of winter temperatures appear to be manifest through both direct and indirect effects. There is a direct negative association between winter temperatures and abundance in the same year (see path coefficients -0.49 and -0.59, Figure 2). Many mechanisms are possible, including desiccation of pupae, leading to mortality and lower adult numbers. In contrast, there is a direct positive association between winter temperature in the previous year and abundance in the subsequent year. This direct effect could be mediated through the larval stage, with warmer conditions in one year being associated with greater plant growth and increased larval survival through the subsequent spring, translating into increased adult numbers in the next year. Roy et al. (2001) similarly found positive associations for a number of butterfly species between temperatures in the previous year and abundance in the subsequent year.

The observed associations with weather coexist with an effect of density dependence (Figure 2) that is generally weaker than the weather effects, but still significant and potentially important for the observed dynamics of the *G. lygdamus* population (Figure 1). It is particularly interesting to note the indirect positive effect that previous winter temperature has on the dynamics of the current year's population (summarized as ΔN) when mediated through the density dependent effect of last year's population; the path from previous winter temperature to previous density is negative, which becomes a positive effect on the current year, ΔN, when combined with the negative connection between the previous year and the current year. A role for density dependence is not always considered in studies of insect population dynamics and weather, perhaps due to a perception that insects might be more often regulated by external factors (Nowicki et al. 2009). The results reported here are a reminder that density dependence should not be neglected even in highly fluctuating invertebrate populations.

We have suggested hypotheses to explain phenological patterns that could be tested with observational or experimental data from other *G. lygdamus* populations. Lacking other ecological data, such as abundance of nectar or predators, and data from other *G. lygdamus* populations, definitive conclusions cannot be drawn about the causes behind the colonization and extinction of our focal population. Fluctuations in precipitation and temperature certainly played an important role, however, in the population fluctuations and extinction of the population. The open diamonds in Figure 1 correspond to the predicted values from our top multiple regression model (Table 1), which illustrates the good fit of our model in general and indicates years in which our model would have predicted extinction for the population. Of course the model is not perfect, and it is interesting to note that the year after the last butterfly was observed was predicted to be a rebound year (Figure 1). Sensitivity to precipitation has been implicated in other extinctions of butterfly populations (Ehrlich et al. 1980), including the extinction of a montane *G. lygdamus* population that was anecdotally attributed to a single snowstorm (Ehrlich et al. 1972). The results reported here are also consistent with other recent studies of butterflies that have found the importance of density dependence to be comparable to the importance of abiotic conditions in explaining variation in abundance (e.g. Nowicki et al. 2009).

In summary, we found a complex relationship between butterfly abundance and weather that poses a serious challenge both for conservation and predictions associated with global climate change. As California, for example, gets warmer and drier (Ackerly et al. 2010), it is unclear what impact these changes will have on *G. lygdamus* populations. The positive association with precipitation might lead to population declines in a drier region, or more complex associations (possibly mediated through density dependence) with temperature might somehow compensate. While there is much to learn, these results and other fine-scale studies of butterfly populations reveal a level of complexity that complements broader and more regional analyses of threats and declines (Thomas and Abery 2004; Thomas et al. 2004; Parmesan 2006; Forister et al. 2010).
